# Supplementary material for: Kinetochore-bound Mps1 regulates kinetochore–microtubule attachments via Ndc80 phosphorylation
Source: J Cell Biol. 2021 Oct 14;220(12):e202106130. doi: 10.1083/jcb.202106130 (PMC8641409; doi:10.1083/jcb.202106130)
Supplement: Table S1 — lists yeast strains used in this study. [file JCB_202106130_TableS1.docx]

**Supplemental Table 1.** Strains used in this study.

All strains are derivatives of SBY3 (W303)

**Strain Relevant Genotype**

| SBY3 (W303) | *MAT*a *ura3-1 leu2-3,112 his3-11 trp1-1 can1-100 ade2-1 bar1-1* |
| --- | --- |
| SBY8253 | *MATa DSN1-6His-3Flag:URA3* |
| SBY8712 | *MATa DSN1-6His-3Flag:URA3 ipl1-321* |
| SBY8726 | *MATa DSN1-6His-3Flag:URA3 mps1-1* |
| SBY9453 | *MATa pNDC80-ndc80-14D-3HA:KanMX NDC80:URA3(CEN plasmid)* |
| SBY10315 | *MATa DSN1-6His-3Flag:URA3 spc105Δ:TRP*  *his3::pSPC105-spc105-6A-12Myc:HIS3* |
| SBY10336 | *MATa DSN1-6His-3Flag:URA3 spc105Δ:TRP*  *his3::pSPC105-SPC105-12Myc:HIS3* |
| SBY11334 | *MATa pNDC80-ndc80-14D-3HA:KanMX mad3∆::HIS3*  *NDC80:URA3(CEN plasmid)* |
| SBY11808 | *MATa DSN1-6His-3Flag:URA3 NDC80-3HA:KanMX* |
| SBY13916 | *MATa DSN1-6His-3Flag:URA3 STU2-3HA-IAA7:KanMX*  *leu2::pSTU2-STU2-3V5:LEU2 his3::pGPD1-OsTIR1:HIS3* |
| SBY15087 | *MATa pNDC80-ndc80-14D-3HA:KanMX mad2∆::KanMX*  *NDC80:URA3(CEN plasmid)* |
| SBY15139 | *MATa pNDC80-ndc80-14D-3HA:KanMX bub1∆::HphMX*  *NDC80:URA3(CEN plasmid)* |
| SBY15149 | *MATa pNDC80-ndc80-14D-3HA:KanMX trp1::pNDC80-NDC80-3V5-IAA7:TRP1 his3::pGPD1-OsTIR1:HIS3 NDC80:URA3(CEN plasmid)* |
| SBY17527 | *MATa STU2-3V5-IAA7:KanMX trp1::pGPD1-OsTIR1:TRP1 CEN8::lacO:TRP1 ura3::TUB1-CFP:URA3 his3::pCUP1-GFP-LacI:HIS3 leu2::pSTU2-STU2-3V5:LEU2* |
| SBY17624 | *MATa NDC80-3HA:KanMX mad2∆::HIS3 PDS1-18Myc:LEU2* |
| SBY17648 | *MATa NDC80-3HA:KanMX PDS1-18Myc:LEU2* |
| SBY17668 | *MATa stu2-3V5-IAA7:KanMX trp1::pGPD1-OsTIR1:TRP1 CEN8::lacO:TRP1 ura3::TUB1-CFP:URA3 his3::pCUP1-GFP-LacI:HIS3*  *leu2::pSTU2-STU2-3V5:LEU2 mad3∆::NatMX* |
| SBY17807 | *MATa PDS1-18Myc:LEU2 ndc80-14A-3HA:KanMX* |
| SBY17895 | *MATa PDS1-18Myc:LEU2 mad2∆::HIS3 ndc80-14A-3HA:KanMX* |
| SBY17991 | *MATa pNDC80-ndc80-14D-3HA:KanMX bub3∆::HphMX*  *NDC80:URA3(CEN plasmid)* |
| SBY17993 | *MATa pNDC80-ndc80-14D-3HA:KanMX mad1∆::HIS3*  *NDC80:URA3(CEN plasmid)* |
| SBY18446 | *MATa NDC80-3HA:KanMX NDC80:URA3(CEN plasmid)* |
| SBY19186 | *MATa DSN1-6His-3Flag:URA3 ndc80Δ:NatMX trp1::pNDC80-NDC80-3HA:TRP1* |
| SBY19380 | *MATa DSN1-6His-3Flag:URA3 ndc80-11A-3HA:KanMX* |
| SBY19817 | *MATa ndc80Δ:NatMX trp1::pNDC80-ndc80-8D-3HA:TRP1* |
| SBY19855 | *MATa DSN1-6His-3Flag:URA3 ndc80Δ:NatMX*  *trp1::pNDC80-ndc80-8A-3HA:TRP1* |
| SBY19877 | *MATa DSN1-6His-3Flag:URA3 ndc80Δ:NatMX*  *trp1::pNDC80-ndc80-8D-3HA:TRP1* |
| SBY20062 | *MATa NDC80-3HA:TRP1* |
| SBY20063 | *MATa ndc80-8A-3HA:TRP1* |
| SBY20170 | *MATa DSN1-6His-3Flag:URA3 STU2-3HA-IAA7: leu2::pSTU2-stu2(∆658-761::GDGAGL^linker^)-3V5:LEU2 his3::pGPD1-OsTIR1:HIS3 NDC80-3HA:TRP1* |
| SBY20171 | *MATa DSN1-6His-3Flag:URA3 STU2-3HA-IAA7:KanMX*  *leu2::pSTU2-stu2(∆658-761::GDGAGL^linker^)-3V5:LEU2 his3::pGPD1-OsTIR1:HIS3 ndc80-8A-3HA:TRP1* |
| SBY20199 | *MATa leu2::pGPD1-OsTIR1:LEU2 trp1::pNDC80-NDC80-3HA:TRP1*  *NDC80-3V5-IAA7:KanMX dam1-3D(S257D,S265D,S292D):KanMX* |
| SBY20201 | *MATa leu2::pGPD1-OsTIR1:LEU2 trp1::pNDC80-ndc80-8D-3HA:TRP1*  *NDC80-3V5-IAA7:KanMX dam1-3D(S257D,S265D,S292D):KanMX* |
| SBY20210 | *MATa STU2-3V5-IAA7:KanMX trp1::pGPD1-OsTIR1:TRP1 CEN8::lacO:TRP1 ura3::TUB1-CFP:URA3 his3::pCUP1-GFP-LacI:HIS3 mad3∆::NatMX*  *leu2::pSTU2-stu2(∆658-761::GDGAGL^linker^)-3V5:LEU2 NDC80-3HA:TRP1* |
| SBY20211 | *MATa STU2-3V5-IAA7:KanMX trp1::pGPD1-OsTIR1:TRP1 CEN8::lacO:TRP1 ura3::TUB1-CFP:URA3 his3::pCUP1-GFP-LacI:HIS3 mad3∆::NatMX*  *leu2::pSTU2-stu2(∆658-761::GDGAGL^linker^)-3V5:LEU2 ndc80-8A-3HA:TRP1* |
| SBY20217 | *MATa NDC80-3V5-IAA7:KanMX trp1:pNDC80-ndc80-8D-3HA:TRP1*  *pGPD-OsTIR1:LEU2 dam1-3D(S257D,S265D,S292D):KanMX* |
| SBY20361 | *MATa DSN1-6His-3Flag:URA3 PDS1-18Myc:LEU2 mad3∆::HIS3 mcd1-1* |
| SBY20362 | *MATa DSN1-6His-3Flag:URA3 PDS1-18Myc:LEU2 mad3∆::HIS3* |
| SBY20622 | *MATa DSN1-6His-3Flag:URA3 PDS1-18Myc:LEU2 mad3∆::HIS3 mcd1-1*  *mps1-1* |
| SBY20893 | *MATa leu2::pGPD1-OsTIR1:LEU2 his3::pCUP1-GFP-LacI:HIS3 CEN8::lacO:TRP1 CDC20-AID:KanMX ndc80-8A-3HA:TRP1* |
| SBY20894 | *MATa leu2::pGPD1-OsTIR1:LEU2 his3::pCUP1-GFP-LacI:HIS3 CEN8::lacO:TRP1 CDC20-AID:KanMX NDC80-3HA:TRP1* |
