# Supplementary material for: Kinetochore-bound Mps1 regulates kinetochore–microtubule attachments via Ndc80 phosphorylation
Source: J Cell Biol. 2021 Oct 14;220(12):e202106130. doi: 10.1083/jcb.202106130 (PMC8641409; doi:10.1083/jcb.202106130)
Supplement: Table S2 — lists plasmids used in this study. [file JCB_202106130_TableS2.docx]

**Supplemental Table 2**Plasmids used in this study

**Plasmid Description Mutations**

| pSB1302 | *pNDC80-NDC80:URA3* (CEN plasmid) | none |
| --- | --- | --- |
| pSB1577 | *pNDC80-ndc80-14D:KanMX6* (pSK981, gift from Lechner lab) | S4D,T5D,S6D,T21D,S22D,S37D,T38DT43D,T74D,T79D,T82D,S205D,T248D,T252D |
| pSB1848 | *pNDC80-ndc80-14A:KanMX6* (pSK1039, gift from Lechner lab) | S4A,T5A,S6A,T21A,S22A,S37A,T38A,T43A,T74A,T79A,T82A,S205A,T248A,  T252A |
| pSB2412 | *pNDC80-NDC80-3HA:TRP1* | none |
| pSB3131 | *pNDC80-ndc80-11A-3HA:KanMX6* (derivative of pSB1848) | S4A,T5A,S6A,S22A,T38A,T43A,T79A,  T82A,S205A,T248A,T252A |
| pSB3207 | *pNDC80-ndc80-8A-3HA:TRP1* (derivative of pSB2412) | S4A,T5A,S6A,S22A,T38A,T43A,T79A,  T82A |
| pSB3208 | *pNDC80-ndc80-8D-3HA:TRP1* (derivative of pSB2412) | S4D,T5D,S6D,S22D,T38D,T43D,T79D,T82D |
