# Supplementary material for: Kinetochore-bound Mps1 regulates kinetochore–microtubule attachments via Ndc80 phosphorylation
Source: J Cell Biol. 2021 Oct 14;220(12):e202106130. doi: 10.1083/jcb.202106130 (PMC8641409; doi:10.1083/jcb.202106130)
Supplement: Table S3 — lists primers used in this study. [file JCB_202106130_TableS3.docx]

**Supplemental Table 3.**

Primers used in this study (all listed 5' to 3')

**Primers Sequence**

| SB4443 | GATCGATCtctagaTCAGCACTGAGCAGCGTAATCTGGAACG |
| --- | --- |
| SB4732 | GATCGATCgggcccGGTCTCTGTAGGGTCAATAG |
| SB4877 | CCAGTTTACCTGGCCATTCTTGACTCTTTTGCTTCATCGCGTTGAC |
| SB5014 | CACAATTGAGGAGAAGAAACtCCGCGAATCAAGGCCTAGCCG |
| SB7001 | ATCGATCGGGTTACCCATACTGAAAGTGCCAAAAGAAAAAAAGATAACGTATGGAATGATATAGTCACAAAACTAATATTTCCTAAAATAAACAAACAACACCAAAAATGGTAGAAATAATTGGAATACATTCACAGGAGAGGTAGAATCGTCCCTGTAAAAAAAATTGAAACGGAACAAGAAAAATAACTGACGTTCCTTTCTTTAAACGTTAGGGTGTACTATAATATATTCCATAAGAGATACCCGTTTCTATAAATGCAAAGCGACGATGACACTGATCAACATGTGCTACATCACATGGACCCTCATCGGTTTACGGATCAAATACCAACTGCAACATCGTCACAATTGAGGAGAAGAAACAGCGACAATCAAGGTCTAGATGACATGATCAATAAGAGTATTGCCAGGAATACAATAAGCGGGACTGGCATTCCCACAGGAGGCATAAATAAAAATAAGAGGACAAGAAGCACGGTTGCAGGAGGTGACAATGGTGATGCATTGGCTCTAAATGACAAATCCAACAGTAGAAACAGCGTCAGCAGATTATCAATAAATCAACTTGGCAGCCTGCAGCAACATCTGAGCAATAGAGATCCAAGGCCACTAAGAGACAAAAACTTCCAAAGCGCTATTCAAGAGGAGATTTATGACTATTTGAAAAAGAATAAATTTGATATTGAAACAAATCATCCCATTTCAATAAAATTTCTAAAACAACCCACTCAAAAGGGGTTTATTATCATTTTCAAGTGGTTATATTTAAGACTCGATCCAGGTTACGGCTTTACTAAGTCTATCGAGAATGAGATCTATCGATCG |
| SB7002 | ATCGATCGGGTTACCCATACTGAAAGTGCCAAAAGAAAAAAAGATAACGTATGGAATGATATAGTCACAAAACTAATATTTCCTAAAATAAACAAACAACACCAAAAATGGTAGAAATAATTGGAATACATTCACAGGAGAGGTAGAATCGTCCCTGTAAAAAAAATTGAAACGGAACAAGAAAAATAACTGACGTTCCTTTCTTTAAACGTTAGGGTGTACTATAATATATTCCATAAGAGATACCCGTTTCTATAAATGCAAAGCGCAGCAGCTACTGATCAACATGTGCTACATCACATGGACCCTCATCGGTTTACGGCCCAAATACCAACTGCAACATCGTCACAATTGAGGAGAAGAAACAGCGCGAATCAAGGTCTAGCCGACATGATCAATAAGAGTATTGCCAGGAATACAATAAGCGGGACTGGCATTCCCACAGGAGGCATAAATAAAAATAAGAGGACAAGAAGCACGGTTGCAGGAGGTGCAAATGGTGCAGCATTGGCTCTAAATGACAAATCCAACAGTAGAAACAGCGTCAGCAGATTATCAATAAATCAACTTGGCAGCCTGCAGCAACATCTGAGCAATAGAGATCCAAGGCCACTAAGAGACAAAAACTTCCAAAGCGCTATTCAAGAGGAGATTTATGACTATTTGAAAAAGAATAAATTTGATATTGAAACAAATCATCCCATTTCAATAAAATTTCTAAAACAACCCACTCAAAAGGGGTTTATTATCATTTTCAAGTGGTTATATTTAAGACTCGATCCAGGTTACGGCTTTACTAAGTCTATCGAGAATGAGATCTATCGATCG |
