# Supplementary material for: Kinetochore-bound Mps1 regulates kinetochore–microtubule attachments via Ndc80 phosphorylation
Source: J Cell Biol. 2021 Oct 14;220(12):e202106130. doi: 10.1083/jcb.202106130 (PMC8641409; doi:10.1083/jcb.202106130)
Supplement: Table S4 — summarizes laser trap results. [file JCB_202106130_TableS4.docx]

**Supplemental Table 4. Summary of laser trap results.**

Rupture forces indicate median ± σ obtained from bootstrapping of N individual rupture events, with replacement. All the individual rupture force values are provided in Supplemental Table S5.

| **Figure 1 & S1** | **Strain Number** | **Kinetochore Type, Treatment** | **[Dsn1] (nM)** | **Median Rupture Force (pN) (*N*)** |
| --- | --- | --- | --- | --- |
|  | SBY8253 | wild type, no treatment | 2 | 9.83 ± 0.50 (*156*) |
|  |  | wild type, ADP-treated | 2 | 8.66 ± 0.56 (*110*) |
|  |  | wild type, ATP-treated | 2 | 5.31 ± 0.56 (*118*) |
|  |  | wild type, ATP- & Phosphatase-treated | 2 | 7.53 ± 0.62 (*69*) |
|  |  | wild type, MnCl_2_-treated | 2 | 8.57 ± 1.45 (*37*) |
|  |  | wild type, MnCl_2_- & ADP-treated | 2 | 8.08 ± 0.84 (*52*) |
|  |  | wild type, MnCl_2_- & ATP-treated | 2 | 4.84 ± 1.12 (*24*) |

| **Figure 2** | **Strain Number** | **Kinetochore Type, Treatment** | **[Dsn1] (nM)** | **Median Rupture Force (pN) (*N*)** |
| --- | --- | --- | --- | --- |
|  | SBY8253 | wild type, DMSO-treated | 2 | 8.15 ± 0.64 (*85*) |
|  |  | wild type, DMSO- & ADP-treated | 2 | 8.20 ± 0.44 (*80*) |
|  |  | wild type, DMSO- & ATP-treated | 2 | 4.97 ± 0.39 (*100*) |
|  |  | wild type, DMSO- & Reversine-treated | 2 | 8.11 ± 0.66 (*74*) |

| **Figure 3 & S2** | **Strain Number** | **Kinetochore Type, Treatment** | **[Dsn1] (nM)** | **Median Rupture Force (pN) (*N*)** |
| --- | --- | --- | --- | --- |
|  | SBY10315 | Spc105-6A (Myc), no treatment | 4 | 8.34 ± 0.38 (*84*) |
|  |  | Spc106-6A (Myc), ADP-treated | 4 | 8.66 ± 0.60 (*57*) |
|  |  | Spc105-6A (Myc), ATP-treated | 4 | 3.17 ± 0.25 (*59*) |
|  | SBY19380 | Ndc80-11A (3HA), no treatment | 3 | 8.66 ± 0.64 (*96*) |
|  |  | Ndc80-11A (3HA), ADP-treated | 3 | 10.04 ± 0.98 (*74*) |
|  |  | Ndc80-11A (3HA), ATP-treated | 3 | 8.53 ± 0.62 (*71*) |
|  | SBY11808 | Ndc80 (3HA), no treatment | 3 | 9.92 ± 1.63 (*46*) |
|  |  | Ndc80 (3HA), ADP-treated | 3 | 8.87 ± 2.46 (*37*) |
|  |  | Ndc80 (3HA), ATP-treated | 3 | 5.66 ± 0.46 (*55*) |

| **Figure 5** | **Strain Number** | **Kinetochore Type, Treatment** | **[Dsn1] (nM)** | **Median Rupture Force (pN) (*N*)** |
| --- | --- | --- | --- | --- |
|  | SBY19855 | Ndc80-8A (3HA), no treatment | 4 | 9.36 ± 0.71 (*111*) |
|  |  | Ndc80-8A (3HA), ADP-treated | 4 | 8.64 ± 0.54 (*79*) |
|  |  | Ndc80-8A (3HA), ATP-treated | 4 | 9.41 ± 0.48 (*132*) |
|  | SBY19877 | Ndc80-8D (3HA), no treatment | 4 | 5.60 ± 0.37 (*135*) |
